# Pseudothrombophlebitis syndrome in a rheumatoid arthritis patient with swollen calf and persistent itching: a case report

**DOI:** 10.1186/s12891-018-2263-8

**Published:** 2018-09-24

**Authors:** Cevriye Mülkoğlu, Zeynep Alpoğuz Yılmaz, Barış Nacır, Hakan Genç

**Affiliations:** Department of Physical Therapy and Rehabilitation, Ankara Health Application and Research Hospital, Ulucanlar street, 06230 Altındağ/Ankara, Turkey

**Keywords:** Baker’s cyst, Pseudothrombophlebitis, Rheumatoid arthritis, Musculoskeletal ultrasound

## Abstract

**Background:**

Baker’s cyst is a benign lesion that results from degenerative or inflammatory diseases of the knee joint. When Baker’s cyst ruptures, it may simulate deep vein thrombosis known as Pseudothrombophlebitis syndrome with calf pain, swelling and redness. Pseudothrombophlebitis syndrome without thrombus in popliteal veins has distinct treatment choice than deep vein thrombus.

**Case presentation:**

In this report, we presented a 47 year-old male rheumatoid arthritis patient with complaints of redness, pain and swelling on his right calf. Pseudothrombophlebitis syndrome was diagnosed due to ruptured Baker’s cyst.

**Conclusions:**

We used musculoskeletal ultrasound for both differential diagnosis and treatment of pseudothrombophlebitis syndrome. Ultrasonography revealed massive fluid collection within muscle layers. 280 cc inflammatory fluid was aspirated simultaneously. We also emphasized the importance of ultrasonography in diagnosis and treatment of Pseudothrombophlebitis syndrome with this report.

## Background

Rheumatoid arthritis (RA) is a common chronic autoimmune joint disease which affects females 3 times more. Synovial, bursal, tendon sheath and capsule hypertrophy due to chronic inflammation lead to increasing inflammatory fluid and cause intra-articular pannus formation [1]. Baker’s cyst is a bening nonvascular lesion in popliteal fossa. The expanding semimembranosus-gastrocnemius bursa associated with the knee joint diseases is responsible for Baker’s cysts. Mostly Baker’s cysts are asymptomatic. If Baker’s cyst ruptures it can simulate the symptoms of acute deep vein thrombosis including painful calf swelling, erythema and tenderness on the calf. The clinical feature of deep venous thrombosis and pseudothrombophlebitis syndrome is difficult to distinguish by clinical examination and further imaging techniques are necessary. Magnetic resonance imaging (MRI) and musculoskeletal ultrasound (MUS) are the main imaging modalities for identification of ruptured Baker’s cysts [2].

In this report, we present a Pseudothrombophlebitis syndrome in a RA patient due to ruptured Baker’s cyst. This condition was diagnosed and treated rapidly and simultaneously with ultrasound. The swollen calf of patient was evaluated by MUS and synovial fluid collection within the gastrocnemius muscle layers was drained from multiple points.

## Case presentation

A 47-year-old male admitted to our department with pain and swelling on his right calf. The patient was diagnosed with RA 5 years ago. He had no history of knee trauma. On physical examination, tenderness of wrists and elbows, swelling on the left wrist and contracture of right elbow were found. McMurray test was negative bilaterally. There was no swelling in the left knee. Right knee flexion was limited and Ballotman test was positive. There was bilaterally knee joint tenderness with palpation.

**Fig. 1:**
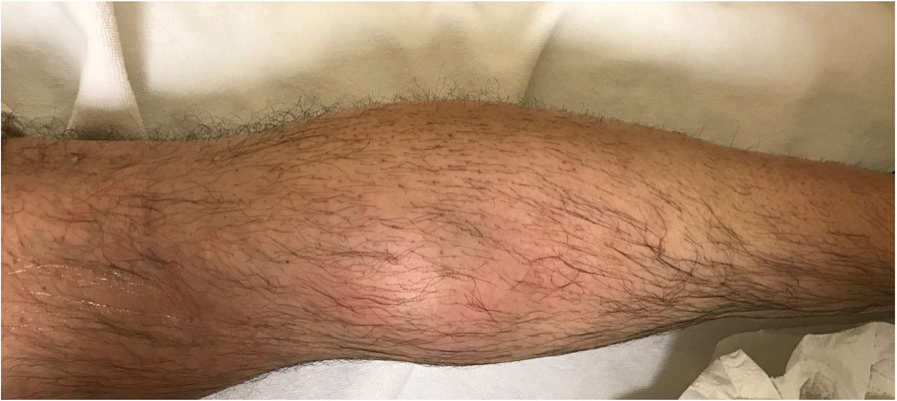
There was swelling and redness on patient’s calf and was similar to deep vein thrombophlebitis

He had painful swelling and redness on his right calf (Fig. 1). There was a persistent itching on the skin of the right calf. Homans test was positive on the right. Plain radiographs showed mild degenerative changes at knee joints. Erythrocyte sedimentation rate was 22 mm/hour, C-reactive protein was 24.7 mg/L, WBC count was 14350, rheumatoid factor was positive. Complete blood count except for WBC and biochemical laboratory tests were within normal limits.

**Fig. 2:**
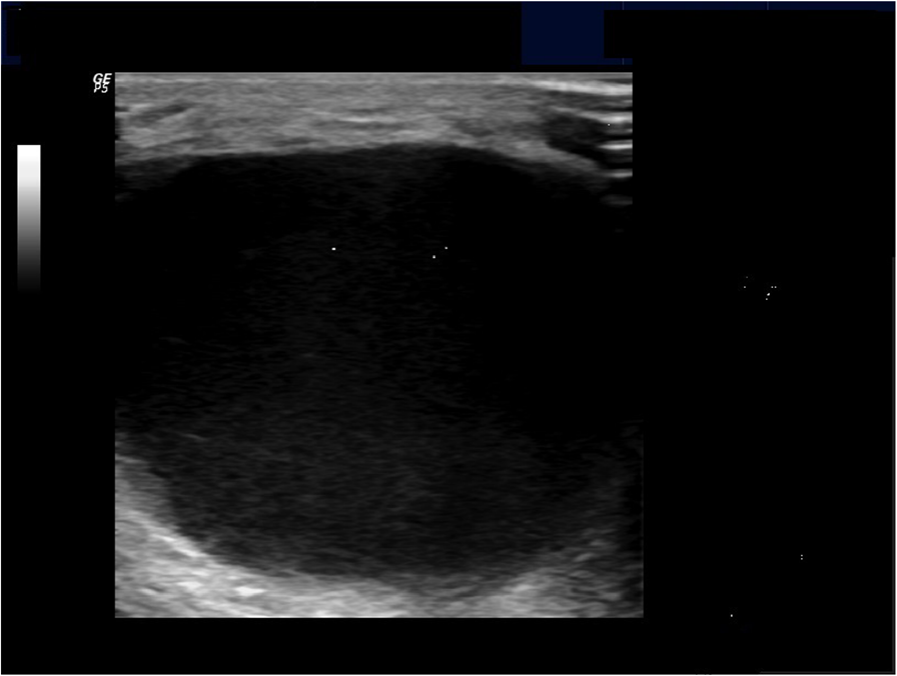
There was a huge fluid collection between fascial compartments and muscle layers on ultrasonography

These findings on the calf were similar to deep vein thrombosis. We used ultrasonography for differential diagnosis of deep vein thrombosis. Ultrasonography revealed massive fluid collection within the fascial compartments and gastrocnemius muscle layers (Fig. 2). There were two septas between fluid collections. Color Doppler ultrasonography showed normal flow pattern in popliteal veins. According to these findings, the patient was diagnosed as Pseudothrombophlebitis syndrome due to rupture of Baker’s cyst. 280 cc inflammatory fluid was aspirated from three different points under the guidance of ultrasonography. After the intervention, the swelling on the right calf decreased. The patient was relieved and elastic bandage was applicated to his calf. After 1 week follow-up, the swelling on his calf repeated and another intervention was performed. Approximately 100 cc inflammatory fluid was aspirated and 1 cc steroid (betamethasone) was injected into right knee joint. After 1 month follow-up, we found that the pain and redness on his calf was clearly decreased. The patient’s pain visual analog scale (VAS) value was decreased from 8 to 2. We observed that the patient continued to improve at follow-up visit 3 months later; VAS value was 0 and there was no swelling or redness on his calf.

## Discussion

Rheumatoid arthritis is a common, chronic, autoimmune inflammatory joint disease. It appears in 3% of females and 1% of males. Chronic inflammation results in neutrophil infiltration in soft tissues and hypertrophy of capsule, synovial, bursa and tendon sheath. Synovial proliferation is associated with increased joint fluid in these patients [1]. RA damages usually small joints such as hands and feet but also wrist, shoulder, elbow, hip, knee and ankle can be affected [3]. Knee joints are involved in 15% of RA patients [1]. In our patient, hands, wrists, elbows and knees were affected. There was a ruptured cystic formation in our patient’s knee associated with increased synovial fluid production.

Popliteal cysts or Baker’s cysts are nonvascular bening lesion on popliteal fossa. Baker’s cysts commonly occur due to increased intra articular fluid following inflammatory and degenerative diseases of the knee joint. But, it can also occur in normal knee joints [4]. Baker’s cysts are usually associated with osteoarthritis, rheumatoid arthritis, trauma, meniscal tears, gout, less commonly infections and spondyloarthropathy or Behçet’s syndrome. Cyst formation occurs because of enlargement of the gastrocnemio-semimembranosus bursa with increased synovial fluid. Baker’s cyst usually appears with swollen soft tissue at posteromedial of the knee. The incidence is higher in osteoarthritic knees and older patients. Mostly Baker’s cysts are asymptomatic and do not require treatment. Bedrest, analgesia, cold application, intra-articular steroid injection, aspiration of the cyst may be tried in symptomatic Baker’s cysts. In rare cases, cyst excision can be performed surgically [5].

When Baker’s cyst ruptures into the gastrocnemius muscle layers it can simulate the symptoms of deep vein thrombosis including acute pain, swelling, redness and tenderness on the calf. There was a ruptured Baker’s cyst in our patient’s calf associated with increased synovial fluid production. This condition is known as pseudothrombophlebitis syndrome without vascular pathology. All of these findings such as swelling leg, redness and tenderness of the calf were present in our patient. Additionally, there was a persistent itching and scratching on his calf skin. Persistent itching may result from irritation of degraded blood products and inflammatory synovial fluid [6].

Therefore, clinical symptoms of deep vein thrombosis and pseudothrombophlebitis syndrome are difficult to distinguish only by physical examination. Further imaging techniques are necessary for differential diagnosis. Because, the treatment of deep vein thrombosis and pseudotrombophlebitis syndrome are distinctly different. Also, with direct compression of Baker’s cyst to popliteal veins or arteries, deep vein thrombosis and Baker’s cyst may be accompanied together [5].

Arthrography and venography is gold standart for diagnosis of deep vein thrombosis, but it is invasive [6, 7]. MRI and ultrasonography are the most important non-invasive imaging methods to evaluate of Baker’s cyst [1]. Color doppler ultrasonography noninvasively rule out deep vein thrombosis [6]. Recently, musculoskeletal ultrasound is preferred for Baker’s cyst evaluation because of its fast, reliability, cost effectiveness and high sensitivity. Baker’s cyst is seen with hypoechoic or anechoic pattern with ultrasound [8]. We diagnosed rapidly a ruptured Baker’s cyst with musculoskeletal ultrasound which was available in our clinic. Additionally, there were two septas between fluid collections in our patient and inflammatory fluid was aspirated from three different regions. If the aspiration process has not been do under ultrasound guidance, whole fluid accumulation could not be aspirated entirely.

It is extremely important to diagnose correctly the Pseudothrombophlebitis syndrome or deep vein thrombosis because of the treatments are quite different. Musculoskeletal ultrasound may be used to distinguish both disorders. If thrombolytic agents or anticoagulants are given for Baker’s cyst assuming deep vein thrombosis, compartment syndrome may develop due to cyst hemorrhage or hematoma formation [5, 7, 8]. Therefore, it is very important to diagnose correctly these two conditions, which are very similar clinically.

## Conclusions

Ruptured Baker’s cyst may mimic deep vein thrombosis. Although the clinical findings are similar, the treatment protocols are very different of these disorders. So, it needs to be diagnosed correctly. In this report, we presented a pseudothrombophlebitis syndrome in a rheumatoid arthritis patient due to ruptured Baker’s cyst. Diagnosis of Baker’s cysts with musculoskeletal ultrasound is more rapid, cheaper and easier. Recently, musculoskeletal ultrasound is providing rapid and noninvasively diagnosis of Baker’s cyst and other joint and soft tissue pathologies such as effusion, enthesopathy, tendinitis in rheumatological patients.

## Abbreviations

MRI: Magnetic resonance imaging
MUS: Musculoskeletal ultrasound
RA: Rheumatoid arthritis
